# Thoracic Endovascular Aortic Repair for Kommerell’s Diverticulum

**DOI:** 10.3400/avd.oa.25-00091

**Published:** 2025-11-22

**Authors:** Masato Hayama, Go Kuwahara, Hiromitsu Teratani, Mau Amako, Hiroyuki Ito, Hideichi Wada

**Affiliations:** 1 Department of Cardiovascular Surgery, Fukuoka University Hospital, Fukuoka, Fukuoka, Japan; 2 Cardiovascular Surgery, Hakujyuji Hospital, Fukuoka, Fukuoka, Japan; 3 Vascular Surgery, Saiseikai Fukuoka General Hospital, Fukuoka, Fukuoka, Japan

**Keywords:** thoracic endovascular aortic aneurysm, Kommerell’s diverticulum, TEVAR

## Abstract

**Objectives:**

Thoracic endovascular aortic repair (TEVAR) has recently emerged as a less invasive alternative to open thoracotomy for the treatment of Kommerell’s diverticulum (KD). However, anatomical challenges, including an acute aortic arch and an aberrant subclavian artery, often limit its feasibility. This study aimed to evaluate the outcomes of TEVAR for KD.

**Methods:**

Between February 2012 and July 2023, 6 patients with KD underwent TEVAR at 3 institutions. Subclavian artery embolization or reconstruction was performed when necessary. Morphological parameters, including the Kommerell’s diverticulum diameter (KDd) and the distance to the opposite aortic wall (DAW), were assessed.

**Results:**

Four patients underwent subclavian artery embolization, including 1 requiring bilateral embolization. Subclavian artery reconstruction was performed in 2 cases. Intraoperative type 1a endoleaks were observed in 3 cases and were successfully managed with additional stent grafts. During a follow-up period ranging from 13 to 83 months, 1 patient required open surgical conversion due to graft infection. No other severe complications or aneurysmal progression were noted.

**Conclusions:**

Despite the limited follow-up period and lack of long-term data, TEVAR for KD demonstrated favorable short- to mid-term outcomes and may represent an effective treatment option in selected patients.

## Abbreviations

TEVAR
: thoracic endovascular aortic repair
KD
: Kommerell’s diverticulum
SG
: stent graft
DAW
: distance to the opposite aortic wall
KDd
: Kommerell’s diverticulum diameter
RTAD
: right-sided thoracic aortic dissection

## Introduction

Kommerell’s diverticulum (KD) is a congenital anomaly resulting from incomplete regression of the dorsal aorta during the 6th to 7th week of gestation. It was first described by Burckhardt F. Kommerell in 1936.^1)^ KD is defined as a diverticular outpouching at the proximal descending portion of the aortic arch, typically associated with an aberrant origin of the subclavian artery. Variations in aortic arch anatomy—including left- or right-sided aortic arches and anomalous subclavian artery origins—are established during fetal development due to abnormal branching of the aortic arch.

Approximately 0.7%–2.0% of children are born with a left-sided aortic arch and a right aberrant subclavian artery, and 0.04%–0.4% with a right-sided aortic arch and left aberrant subclavian artery. Among these, 20%–60% develop KD.^2,3)^ Due to the rarity of this condition, large-scale case series are lacking, and standardized treatment strategies have not yet been established.

In recent years, thoracic endovascular aortic repair (TEVAR) has been increasingly reported as a less invasive alternative to open thoracotomy for the treatment of KD. However, the acute-angled aortic arch and the presence of an aberrant subclavian artery often pose anatomical challenges for TEVAR. On the other hand, open surgery is also complicated by these anatomical variations. Given the rarity of KD, published cases of TEVAR for KD remain limited, and further investigation is warranted.

## Patients and Methods

We performed TEVAR for KD in 6 patients at 3 centers between February 2012 and July 2023. Although the anatomy of the partial arch differed in each case, we decided to perform stent grafts (SGs) from the end of the branch artery, more centrally than the KD. If an aberrant subclavian artery arose from the KD, we performed embolization of the subclavian artery and compared the right and left vertebral arteries to determine whether the subclavian artery branching from the KD was dominant. The decision to reconstruct was based on whether the branching vertebral artery was dominant.

In addition, patient age, comorbidities, and prior thoracic surgery were taken into account, and TEVAR was prioritized when the surgical risk of open repair was considered high. Moreover, if patients expressed a strong preference for TEVAR after being informed of its uncertainties, this preference was respected and TEVAR was prioritized. Device selection was left to the discretion of each institution but was generally based on the instructions for use (IFU), with oversizing of approximately 15%–20% of the proximal neck diameter. In cases with an acutely angled arch, slightly greater oversizing was chosen when necessary. Postoperative follow-up consisted of computed tomography angiography (CTA) at 1, 3, and 6 months, at 1 year, and annually thereafter.

**Fig. 1:**
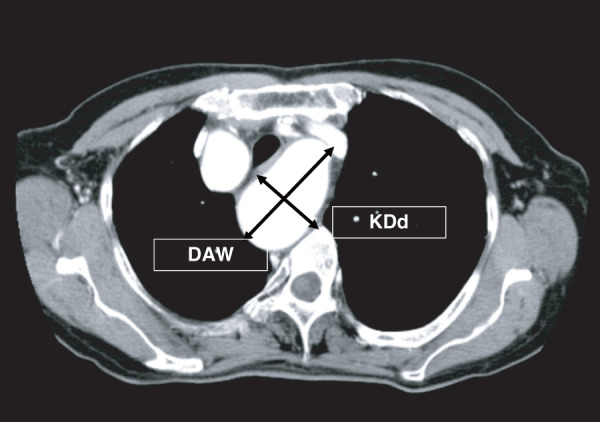
Measurement methods for Kommerell’s diverticulum. DAW: distance to the opposite aortic wall; KDd: Kommerell’s diverticulum diameter

Although there is still no worldwide agreement on the measurement of mass diameter, the distance to the opposite aortic wall (DAW) and the KD diameter (KDd) were mainly used to measure the mass diameter (**Fig. 1**).

## Results

**Table 1** presents the CT findings of the aorta in 6 cases. A right-sided aortic arch was identified in 4 cases. The branch vessels are listed in order of their proximity to the aortic root. The proximal neck length was defined as the length of the normal aortic segment from the tip of the implanted SG, and the proximal neck diameter corresponded to the diameter at the tip of the implanted SG (**Table 1**).

**Table 1:** Computed tomographic findings of each case

Case	Age	Sex	Arch	1st branch	2nd branch	3rd branch	4th branch	DAW (mm)	KDd (mm)	PNL (mm)	PND (mm)
1	68	F	Right	LCCA	RCCA	RSCA	LSCA	62	35	24	31
2	80	M	Left	RCCA	LCCA	LSCA	RSCA	55	27	16	29
3	72	M	Right	RSCA	RCCA	LCCA	LSCA	48	30	43	26
4	67	M	Left	RCCA	LCCA	LSCA	RSCA	81	46	16	28
5	69	M	Right	LCCA	RCCA	RSCA	LSCA	50	38	32	31
6	51	F	Right	LSCA	LCCA	RCCA	RSCA	34	15	34	20

Due to anatomical variations among the cases, the CT findings of each patient are individually presented. RCCA: right common carotid artery; LCCA: left common carotid artery; RSCA: right subclavian artery; LSCA: left subclavian artery; DAW: distance to opposite aortic wall; KDd: Kommerell’s diverticulum diameter; PNL: proximal neck length; PND: proximal neck diameter

**Table 2** summarizes the TEVAR procedures performed in this study. Four patients required subclavian artery embolization, including 1 patient who required bilateral embolization. Subclavian artery reconstruction was performed in 2 patients.

**Table 2:** Case-specific surgical strategies and intraoperative findings

Case	Device	SCA embolization	SCA reconstruction	Intraoperative endoleak
1	cTAG	LSCA	N/A	Type1a endoleak
2	TX2	RSCA	N/A	Type1a endoleak
3	cTAG	N/A	N/A	N/A
4	Valiant	LSCA, RSCA	LSCA-LCCA	N/A
5	Zenith Alpha	LSCA	LSCA-LCCA	Type1a endoleak
6	cTAG	N/A	N/A	N/A

Devices used and surgical procedures are listed, along with intraoperative aortographic findings of endoleaks. LCCA: left common carotid artery; RSCA: right subclavian artery; LSCA: left subclavian artery

**Fig. 2:**
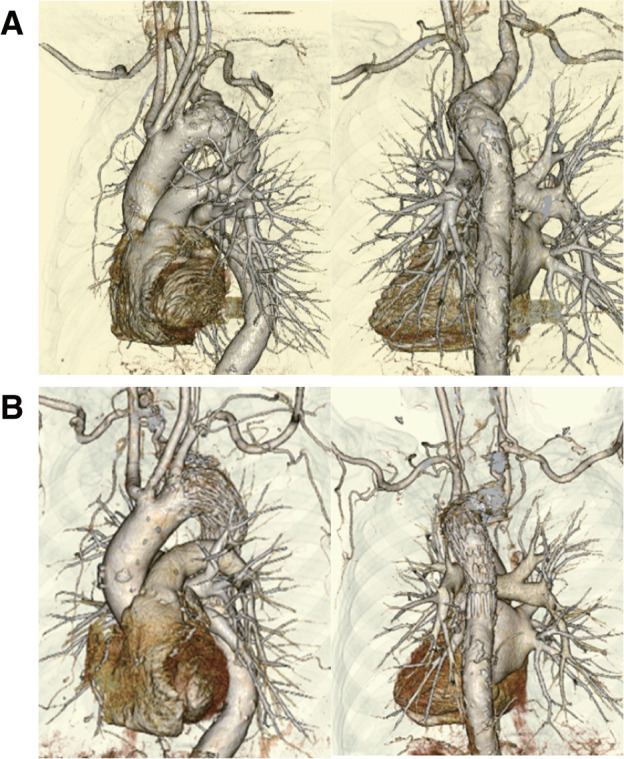
Representative 3D-CTA images of a patient with Kommerell’s diverticulum. (**A**) Preoperative 3D-CTA. (**B**) Postoperative 3D-CTA. 3D: 3-dimensional; CTA: computed tomography angiography

Intraoperative angiography revealed a type 1a endoleak (EL) in 3 patients, all of which were successfully managed with additional SGs. In this series, a type 1a EL occurred in 3 of 6 patients (50%), mainly due to severe arch angulation. Representative pre- and postoperative 3-dimensional CTA images of a patient with a steeply angulated arch are presented in **Fig. 2**.

During follow-up, the aneurysm diameter in Case 1 showed an increase due to a type 2 EL. Coil embolization of the responsible bronchial artery was performed 6 months after surgery, resulting in subsequent aneurysm shrinkage. In Case 4, a postoperative SG infection led to aortic rupture, necessitating open conversion 3 months after the initial procedure. In the remaining cases, no aneurysm enlargement was observed; notably, in Cases 2 and 3, the aneurysms almost completely disappeared. No postoperative complications, including cerebral infarction or major vascular events, occurred during the follow-up period (**Table 3**).

**Table 3:** Postoperative outcomes

Case	Postoperative endoleak	Adjunctive therapy	Aneurysmal diameter change (mm)	Follow-up period	Major event
1	Type 2: bronchial artery	Coil embolization of the responsible bronchial artery	DAW: 45; KDd: 21	72 Months	N/A
2	N/A	N/A	DAW: 45; KDd: 23	83 Months	Subarachnoid hemorrhage 8 years after surgery
3	N/A	N/A	Aneurysmal disappearance	79 Months	N/A
4	N/A	Graft infection; open conversion	N/A	N/A	N/A
5	Type 2: bronchial artery	N/A	DAW: 47; KDd: 32	24 Months	N/A
6	N/A	N/A	DAW: 34; KDd: 15	13 Month	N/A

Postoperative CT findings and subsequent adjunctive therapy are presented. CT findings at short- and mid-term follow-up are also included. CT: computed tomography; DAW: distance to the opposite aortic wall; KDd: Kommerell’s diverticulum diameter; N/A: not applicable

## Discussion

Symptomatic cases are generally considered candidates for intervention; however, the indication for treatment based solely on mass diameter remains undefined. Histopathological studies have demonstrated tunica media necrosis in diverticular lesions,^2,3)^ and aortic rupture and dissection have been reported in 19%^4)^ and 20%^5)^ of cases, respectively. Therefore, therapeutic interventions should be carefully considered. DAW and KDd are the most widely used parameters for surgical decision-making, with DAW >50 mm and KDd >30 mm often cited as surgical thresholds.^6,7)^ However, rupture has been reported even with a KDd of 20 mm,^8)^ and we experienced a case of retrograde type A dissection with a KDd of 19 mm and DAW of 47 mm, as well as an autopsy case with KDd <30 mm and DAW of 50 mm. Notably, ruptured KD cases associated with an aberrant subclavian artery have a 100% mortality rate,^9)^ leading some authors to advocate for aggressive intervention. Thus, determining the indication for surgery based solely on aneurysm diameter remains controversial; however, we recommend considering surgical intervention at the time of KD diagnosis.

Type 1a EL, which is challenging to manage in patients with an acute-angled aortic arch, was observed in 3 cases (Cases 1, 2, and 5). Their proximal neck lengths were 24, 16, and 32 mm, respectively, and all 3 cases exhibited a bird-beak configuration. In each case, the addition of an extra SG successfully eliminated the EL. Although no definitive method exists for the preoperative prediction of bird-beak formation, it is more likely to occur when the landing zone is near the arch apex.^10)^ The use of a proximal bare stent and appropriate oversizing of the proximal neck diameter are known preventive factors.^10)^ In our study, cases in which the SG diameter was approximately 1.3 times the proximal neck diameter avoided type 1a EL, suggesting that adequate oversizing plays an important role in prevention. However, excessive oversizing has been reported to increase the risk of retrograde type A dissection and SG–induced new entry. Therefore, oversizing should generally be limited to around 15%–20%. Oversizing beyond 20%–25% should be reserved only for exceptional cases with extremely acute arches, and oversizing of 30% cannot be recommended.^11)^ Moreover, procedure-specific risks of TEVAR include not only EL but also aortic wall injury, device migration, retrograde dissection, branch ischemia, and spinal cord ischemia. Compared with open surgery, concerns remain regarding the long-term durability of TEVAR, highlighting the importance of careful patient selection and strict lifelong follow-up.

Regarding type 2 EL, 2 patients were affected. One required additional endovascular treatment due to aneurysm enlargement, whereas the other was managed conservatively with serial CT follow-up.

In our series, TEVAR was chosen over open repair in patients with advanced age, significant comorbidities, prior thoracotomy/sternotomy, or when a less invasive approach was strongly preferred by the patient after shared decision-making. These clinical factors, in addition to anatomical feasibility, played a critical role in the decision-making process. While we did not perform a direct comparison with open repair due to the small sample size, previous reports have demonstrated that open and hybrid reconstructions can achieve low perioperative mortality and acceptable long-term durability in experienced centers. In contrast, TEVAR offers lower immediate invasiveness but carries a higher risk of reintervention over time. Thus, our findings should be interpreted in the context of these complementary treatment strategies rather than as evidence of superiority of one approach over another. One of our patients required early reintervention for a persistent type Ia EL, which subsequently resulted in death. This case highlights the vulnerability of endovascular repair in the setting of severe arch angulation and underlines the importance of careful anatomical selection. Moreover, considering the possibility of cumulative reinterventions during long-term follow-up, TEVAR may have inferior durability compared with conventional open repair in selected patients.

Additionally, 3 cases of KD were diagnosed by upper endoscopy based on external esophageal compression. None of these patients had dysphagia or other gastrointestinal symptoms; therefore, open repair—which allows direct mass decompression—was not indicated. If dysphagia or other compression-related symptoms had been present, TEVAR would have been inappropriate. Upper endoscopy should be repeated during follow-up to monitor for the potential development of an aortoesophageal fistula.

A major limitation of this study is the small sample size. Further case accumulation and long-term follow-up are warranted to validate these findings.

## Conclusion

Kommerell’s diverticulum carries risks of rupture and dissection; therefore, therapeutic intervention at the time of diagnosis is recommended. TEVAR can serve as an effective treatment option in selected patients with favorable anatomy or those at high risk for open repair. However, reintervention and long-term durability remain major concerns; therefore, individualized decision-making and rigorous long-term surveillance are essential. Given the small sample size and retrospective nature of this study, these conclusions should be interpreted with caution.

## Additional Remarks

This study was presented at the 52nd Annual Meeting of the Japanese Society for Vascular Surgery (Oita, Japan, May 2024).
